# Data on children׳s neighborhood income trajectories using small geographical units to operationalize neighborhood boundaries

**DOI:** 10.1016/j.dib.2018.10.021

**Published:** 2018-10-10

**Authors:** Tom Kleinepier, Maarten van Ham, Jaap Nieuwenhuis

**Affiliations:** a OTB – Research for the Built Environment, Faculty of Architecture and the Built Environment, Delft University of Technology, The Netherlands; b University of St Andrews, School of Geography and Geosciences, United Kingdom

## Abstract

It is well-known that the spatial scale at which neighborhoods are operationalized can affect the outcomes we observe. This article describes a typology of children׳s neighborhood income trajectories generated by sequence analysis using 100 × 100 m grids to define neighborhoods. The article further describes ethnic differences in the prevalence of the different types of neighborhood trajectories, focusing on the children of the four largest non-Western immigrant groups in the Netherlands (Turks, Moroccans, Surinamese, Antilleans) and native Dutch children. The data can be compared to the research article “*Ethnic differences in timing and duration of exposure to neighborhood disadvantage during childhood*” (Kleinepier et al., 2018).

**Specifications table**

**Table:** 

Subject area	*Social Sciences*
More specific subject area	*Urban Sociology*
Type of data	*Graph and Tables*
How data was acquired	*Data come from the Dutch population register data, referred to as the System of Social statistical Datasets (SSD), hosted by Statistics Netherlands*
Data format	*Analyzed*
Experimental factors	*The data include all Turkish, Moroccan, Surinamese, and Antillean second-generation children who were born in the Netherlands in 1999. In addition, a 5% random sample of native Dutch children born in 1999 was included. The children were observed from birth in 1999 up until age 15 in 2014.*
Experimental features	*Sequence analysis was used to cluster children into a limited number of groups with similar histories of exposure to neighborhood (dis)advantage.*
Data source location	*The Netherlands*
Data accessibility	*Data is with this article*
Related research article	Kleinepier, T., van Ham, M., & Nieuwenhuis, J.G. (2018). Ethnic differences in timing and duration of exposure to neighborhood disadvantage during childhood. Under Review at *Advances in Life Course Research*. [2]

**Value of the data**

• The data presented in this article show ethnic differences in exposure to neighborhood disadvantage in childhood by using a very small spatial scale (i.e., 100 × 100 m grids) to define neighborhood boundaries. This is useful material for research on the modifiable areal unit problem (MAUP).
• The data provide a novel method (sequence analysis) to capture children׳s exposure to neighborhood disadvantage during childhood by simultaneously taking into account the duration and timing of exposure.
• Future research may elaborate on this work by linking the various neighborhood trajectory types to children׳s outcomes in later life. This would shed more light on the relative importance of exposure to neighborhood disadvantage during different developmental stages in childhood (e.g. early childhood vs. adolescence).

## 1 Data

**Fig. 1:**
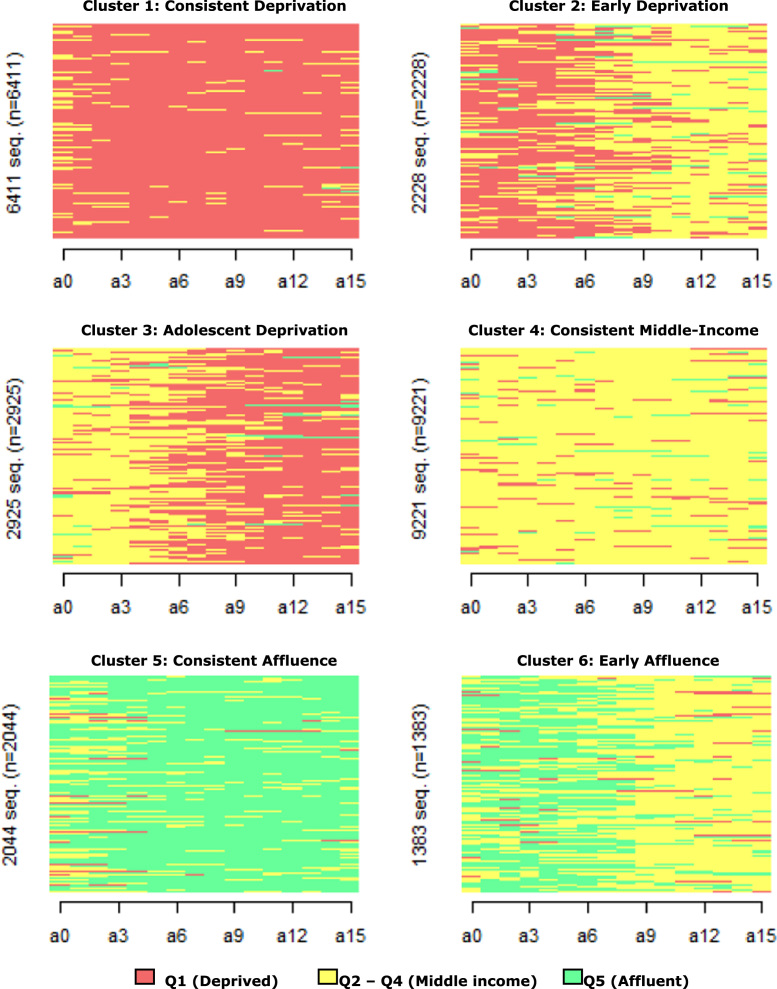
Sequence index plots of six clusters of children׳s neighborhood trajectories using 100 × 100 m grids.

We describe children׳s exposure to neighborhood (dis)advantage during childhood using population register data from the Netherlands [1]. The data in this article can be divided into four parts. In the first part (Fig. 1), we present six different types of neighborhood trajectories in childhood by using sequence index plots. In these plots, each individual is represented by a separate horizontal line. The color of the line indicates the type of neighborhood along chronological age – red for deprived, yellow for middle-income, and green for affluent neighborhoods. The second part of this article (Table 1) compares the typology presented in Fig. 1 to the typology obtained by [2]. In the third part of this article (Table 2, Table 3), we show ethnic differences in the prevalence of the neighborhood trajectory types presented in Fig. 1. Specifically, we compare Turkish, Moroccan, Surinamese, and Antillean second-generation children with native Dutch children. In the fourth and last part of this article (Table 4), we describe ethnic differences in the effect of household income on cluster membership when using 100×100 m grids. Table 2, Table 3, Table 4 may be compared to the results obtained by [2]. This way, it can be observed how ethnic differences in children׳s neighborhood trajectories differ between two spatial scales to define neighborhood boundaries.

**Table 1:** Cross tabulation of the six-cluster typology using 500 × 500 m grids (rows) and 100 ×;100 m grids (columns): Numbers and row percentages (in parentheses).

	*100* ×* 100* *m grids*						
	1	2	3	4	5	6	Total
1. Consistent deprivation	4416 (63.9%)	579 (8.4%)	977 (14.1%)	896 (13.0%)	19 (0.3%)	25 (0.4%)	6912 (100.0%)
2. Early deprivation	418 (22.7%)	603 (32.8%)	192 (10.4%)	568 (30.9%)	31 (1.7%)	26 (1.4%)	1838 (100.0%)
3. Adolescent deprivation	592 (26.2%)	167 (7.4%)	745 (33.0%)	660 (29.2%)	25 (1.1%)	69 (3.1%)	2258 (100.0%)
4. Consistent middle-Income	891 (9.3%)	746 (7.8%)	874 (9.1%)	5843 (60.8%)	607 (6.3%)	655 (6.8%)	9616 (100.0%)
5. Consistent affluence	50 (2.0%)	76 (3.1%)	52 (2.1%)	761 (31.0%)	1188 (48.4%)	328 (13.4%)	2455 (100.0%)
6. Early affluence	44 (3.9%)	57 (5.0%)	85 (7.5%)	493 (43.5%)	174 (15.4%)	280 (24.7%)	1,133 (100.0%)
Total	6411 (26.5%)	2228 (9.2%)	2925 (12.1%)	9221 (38.1%)	2044 (8.4%)	1383 (5.7%)	24,212 (100.0%)

Note: Percentages may not add to 100 due to rounding.

**Table 2:** Percentual distribution over the neighborhood trajectory clusters using 100 × 100 m grids, by ethnicity: Column percentages.

	Turkish (*N* = 5598)	Moroccan (*N* = 5702)	Surinamese (*N* = 4147)	Antillean (*N* = 1367)	Dutch (*N* = 7398)
1. Consistent deprivation	39.2	44.4	18.8	24.5	7.8
2. Early deprivation	10.6	9.1	9.5	10.5	7.8
3. Adolescent deprivation	15.4	15.2	12.4	14.1	6.6
4. Consistent middle-Income	29.9	27.3	41.3	34.8	51.4
5. Consistent affluence	2.3	1.8	10.3	10.8	16.8
6. Early affluence	2.7	2.2	7.9	5.4	9.6
Total	100	100	100	100	100

Note: Percentages may not add to 100 due to rounding.

**Table 3:** Logistic regression analyses of neighborhood trajectory clusters using 100 × 100 m grids on ethnic groups: Logit coefficients. Source: System of Social statistical Datasets (SSD).

	Cluster 1: Consistent deprivation				Cluster 2: Early deprivation				Cluster 3: Adolescent deprivation			
	Model 1a		Model 2a		Model 1b		Model 2b		Model 1c		Model 2c	
	Coef.	SE	Coef.	SE	Coef.	SE	Coef.	SE	Coef.	SE	Coef.	SE
Ethnic group (ref=Dutch)												
Turkish	2.22***	0.05	0.99***	0.06	0.35***	0.06	−0.14	0.08	0.98***	0.06	0.61***	0.08
Moroccan	2.35***	0.05	0.89***	0.07	0.18**	0.06	−0.30***	0.09	0.95***	0.06	0.62***	0.08
Surinamese	1.35***	0.06	0.64***	0.07	0.23**	0.07	−0.23**	0.08	0.77***	0.07	0.47***	0.08
Antillean	1.83***	0.08	0.81***	0.09	0.35**	0.10	−0.17	0.12	0.96***	0.10	0.58***	0.11
Mixed parentage (ref=no)	−0.98***	0.05	−0.58***	0.05	−0.04	0.04	0.00	0.07	−0.23***	0.06	−0.24***	0.06
Father׳s educational level (ref=low/med)												
High			−0.15*	0.06			−0.04	0.07			−0.16*	0.07
Unknown			0.00	0.04			0.13*	0.05			−0.04	0.05
Mother׳s educational level (ref=low/med)												
High			−0.08	0.05			0.00	0.06			−0.06	0.06
Unknown			−0.08*	0.04			−0.01	0.05			−0.09*	0.05
Father׳s labor force participation			−0.15**	0.06			0.33***	0.08				
Mother׳s labor force participation			−0.27***	0.06			0.18*	0.08			0.25***	0.07
Log household income			−1.06***	0.05			−0.18**	0.06			−0.13	0.07
Parents homeowners (ref=rented)			−0.85***	0.05			−0.56***	0.06			−0.58***	0.06
Residential mobility (ref=0 moves)											0.04	0.06
1 move			−0.52***	0.04			0.56***	0.06				
2 moves			−0.60***	0.06			0.76***	0.07			0.35***	0.05
≥3 moves			−0.88***	0.07			0.95***	0.08			0.39***	0.07
Household size			0.18***	0.01			0.13***	0.02			0.76***	0.07
Parental union status (ref=stable union)												
Never lived together			0.09	0.07			0.20*	0.10			0.21*	0.08
Dissolution			−0.07	0.04			−0.23**	0.07			0.30***	0.05
Started living together			0.07	0.09			0.41***	0.11			0.07	0.11
Age difference with father			−0.02***	0.00			−0.00	0.01			−0.00	0.00
Age difference with mother			−0.02***	0.00			−0.02**	0.01			−0.02***	0.01
Constant	−2.48***	0.04	−0.56***	0.15	−2.47***	0.04	−2.77***	0.21	−2.64***	0.05	−1.81***	0.18
Pseudo R^2^	0.13		0.22		0.00		0.03		0.02		0.06	

	Cluster 4: Consistent Middle-Income				Cluster 5: Consistent Affluence				Cluster 6: Early Affluence			
	Model 1d		Model 2d		Model 1e		Model 2e		Model 1f		Model 2f	
	Coef.	SE	Coef.	SE	Coef.	SE	Coef.	SE	Coef.	SE	Coef.	SE
Ethnic group (ref=Dutch)												
Turkish	−1.00***	0.04	−0.51***	0.05	−2.58***	0.10	−0.99	0.12	−1.64***	0.10	−0.53***	0.12
Moroccan	−1.09***	0.04	−0.51***	0.05	−2.65***	0.11	−0.97	0.13	−1.73***	0.10	−0.52***	0.12
Surinamese	−0.59***	0.04	−0.22***	0.05	−1.25***	0.08	−0.48	0.09	−0.71***	0.09	−0.17	0.10
Antillean	−0.93***	0.07	−0.44***	0.04	−1.37***	0.11	−0.59	0.14	−1.24***	0.14	−0.60***	0.15
Mixed parentage (ref=no)	0.42***	0.04	0.25***	0.04	1.27***	0.08	0.72	0.09	0.95***	0.08	0.52***	0.09
												
Father׳s educational level (ref=low/med)												
High			0.06	0.04			0.02	0.07			0.05	0.07
Unknown			0.07*	0.03			−0.10	0.07			−0.19**	0.07
												
Mother׳s educational level (ref=low/med)												
High			−0.03	0.04			0.11	0.07			0.10	0.07
Unknown			0.06*	0.03			0.13	0.07			0.09	0.07
Father׳s labor force participation			0.46***	0.05			0.30*	0.14			0.25	0.14
Mother׳s labor force participation			0.48***	0.05			0.27**	0.09			0.46***	0.10
Log household income			−0.10**	0.04			2.31	0.07			0.57***	0.07
Parents homeowners (ref=rented)			0.37***	0.04			0.39	0.07			0.40***	0.07
												
Residential mobility (ref=0 moves)												
1 move			−0.14***	0.03			0.36	0.06			0.30***	0.07
2 moves			−0.19***	0.05			0.32	0.09			0.55***	0.09
≥3 moves			−0.31***	0.06			0.01	0.12			0.80***	0.11
Household size			−0.07***	0.01			−0.19	0.03			−0.24***	0.03
												
Parental union status (ref=stable union)												
Never lived together			−0.19**	0.07			0.09	0.17			−0.32	0.18
Dissolution			−0.10*	0.04			0.11	0.08			0.19*	0.08
Started living together			−0.11	0.08			−0.21	0.18			0.05	0.17
Age difference with father			0.00	0.00			0.03	0.01			0.01	0.01
Age difference with mother			0.00	0.00			0.06	0.01			0.04***	0.01
Constant	0.06*	0.02	−0.82***	0.13	−1.60***	0.03	−5.14	0.29	−2.24***	0.04	−4.32***	0.30
Pseudo R^2^	0.04		0.06		0.12		0.29		0.06		0.11	

*** *p* <.001.

** *p* < .01.

* *p* <.05.

**Table 4:** Interaction effects between ethnicity and log household income using 100 × 100 m grids: Logit coefficients.

	Consistent deprivation		Consistent middle-Income		Consistent affluence	
	Coef.	SE	Coef.	SE	Coef.	SE
Ethnic group (ref=Dutch)						
Turkish	0.97***	0.07	−0.63***	0.05	−0.85***	0.15
Moroccan	1.00***	0.07	−0.60***	0.05	−0.74***	0.14
Surinamese	0.63***	0.07	−0.41***	0.05	−0.22	0.13
Antillean	0.74***	0.10	−0.62***	0.07	−0.79***	0.21
Log household income (mean centered)	−1.64***	0.11	−0.66***	0.05	2.50***	0.09
HH income × Turkish	0.57***	0.12	1.22***	0.08	−0.25	0.22
HH income × Moroccan	0.95***	0.12	1.07***	0.09	−0.92***	0.22
HH income × Surinamese	0.59***	0.13	0.64***	0.08	−0.51**	0.16
HH income × Antillean	0.39***	0.18	0.60***	0.11	0.33	0.27
Constant	−0.51**	0.15	−0.46**	0.13	−5.36***	0.30
Pseudo R2	0.22		0.07		0.29	

Note: Included are controls for mixed parentage, parental educational level, parental labor force participation, housing tenure, residential mobility, household size, parental union status, and age difference with parents (coefficients not presented).

*** *p* <.001.

** *p* < .01.

## 2 Experimental design, materials and methods

The analyses are based on data from the System of Social statistical Datasets (SSD), which are hosted by Statistics Netherlands. The core of the SSD is the municipal population registers, which provide address information and several demographic characteristics, such as ethnicity, gender, and age. The municipal population registers are linked to other administrative registers, including tax and educational registers. The data are geocoded, indicating the residential neighborhood of each individual at different spatial scales. For the analyses presented in this article, we define neighborhoods as 100 × 100 m grids. We make a selection of ethnic minority children and native Dutch children who are born in 1999. These children are observed over a period of 16 years and their neighborhood status is assessed every year. For each year of observation, we distinguish between three types of neighborhoods: 1. deprived; 2. middle-income; and 3. affluent neighborhoods (see [2] for details).

In order to analyse children׳s neighborhood histories, we make use of sequence analysis. More specifically, using the optimal matching metric, we compute pairwise distances between all sequences (neighborhood trajectories) in the dataset. Subsequently, we use cluster analysis to create groups of children with similar neighborhood histories (for more details, see [2]). The clusters are presented in Fig. 1. In order to estimate ethnic differences in cluster membership, we performed a set of logistic regression analyses, using each of the clusters as the outcome variable. Table 3 includes two different models for each outcome variable. In Model 1, we only include dummy variables for ethnic origin. In Model 2, various parental and household characteristics were added. In Table 4, we interact household income by ethnicity, showing whether the effect of household income differs by ethnicity.
