# Supplementary material for: Data on children׳s neighborhood income trajectories using small geographical units to operationalize neighborhood boundaries
Source: Data Brief. 2018 Oct 10;21:653–9. doi: 10.1016/j.dib.2018.10.021 (PMC6205070; doi:10.1016/j.dib.2018.10.021)
Supplement: Supplementary file 1 — Supplementary material [file mmc1.docx]

Conflicts of Interest

The authors report no conflict of interests.
